# Downregulation of TGF-β1 suppressed proliferation and increased chemosensitivity of ovarian cancer cells by promoting BRCA1/Smad3 signaling

**DOI:** 10.1186/s40659-018-0205-4

**Published:** 2018-12-29

**Authors:** Yanqiu Wang, Jun Xiang, Jianjun Wang, Yazhong Ji

**Affiliations:** 1 Reproductive Medical Center, Department of Gynecology and Obstetrics, Tongji Hospital, Tongji University School of Medicine, Shanghai, China; 2 Department of Gynecology and Obstetrics, Tongji Hospital, Tongji University School of Medicine, Shanghai, China

**Keywords:** Ovarian cancer, TGF-β1, BRCA1, Smad3, Proliferation

## Abstract

**Background:**

Studies have demonstrated that transforming growth factor beta-1 (TGF-β1) exhibits oncogenic activity in different types of cancer, including ovarian cancer (OC). However, its regulatory mechanism in OC and whether TGF-β1 is involved in chemosensitivity regulation remains unclear. Thus, the aim of this study was to investigate the role of TGF-β1 in OC.

**Methods:**

The OC cell line SKOV3 was employed, and TGF-β1 overexpression or knockdown vectors were constructed. The cell proliferation of SKOV3 was evaluated with the cell counting kit (CCK8) kit after treatment with different concentrations of cis-platinum. Western blot and protein immunoprecipitation were employed to detect changes in BRCA1 and Smad3 expression and their interactions. Tumor growth in nude mice was evaluated.

**Results:**

The results showed that TGF-β1 knockdown increased chemosensitivity by promoting BRCA1 expression and Smad3 phosphorylation. In vivo studies showed that TGF-β1 knockdown significantly inhibited the growth of tumors, also by upregulating BRCA1 expression and Smad3 phosphorylation.

**Conclusion:**

Taken together, our results suggest that TGF-β1 knockdown inhibits tumor growth and increases chemosensitivity by promotion of BRCA1/Smad3 signaling.

## Introduction

Ovarian cancer (OC) is the most common and deadly disease among women. The histological divisions include sex cord-stromal, epithelial and germ cell tumors [1–3]. Epithelial OC (EOC) is derived from the fallopian tubes or ovary-originating epithelial cells, with more than 90% of total OC occurring postmenopausally [4, 5]. More than 70% of EOCs are diagnosed in the advanced stage due to the difficulty in detection and the absence of effective diagnostic markers, making it the most deadly gynecological malignant tumor [6, 7]. After surgical intervention, cisplatin is the major chemotherapy drug for OC, but patients tend to develop cisplatin resistance in the clinical setting [8, 9]. Therefore, there is a critical need to identify possible targets for therapeutic intervention.

Transforming growth factor beta (TGF-β) plays an important role in promoting cell proliferation and cell cycle regulation in early oncogenesis and normal tissue development [10, 11]. Previous studies have found that TGF-β1 can activate both SMAD2 and SMAD3 [12]. SMAD2 and 3 dimerize, forming the SMAD2/3 complex. Then, the SMAD2/3 complex interacts with SMAD4 and forms a heterohexameric complex. After translocation into the nucleus, the heterohexameric complex transcriptionally regulates cellular process-related target genes such as the induction of chemotherapy resistance [13–15]. However, it has been shown that patients who have *BRCA1* genetic defects have an increased risk of cancer. Increasing evidence has found that BRCA1 is involved in many cellular processes, including apoptosis, genomic stability, DNA-damage repair and the cell-cycle checkpoint [16]. A previous study found that *BRCA1* expression promotes the interaction of Smad3 and Smad4. Additionally, BRCA1 regulates Smad3-mediated TGF-β signaling by interacting with Smad3 [17]. However, it remains unclear whether the TGF-β signal regulates *BRCA1* expression.

Thus, in this study we investigated the role of TGF-β1 in OC. We also examined whether dysfunction of TGF-β1 leads to decreased growth and increased chemosensitivity by induction of the BRCA1/SMAD3 pathway.

## Materials and methods

### Animals and ethics statement

Five to six week old male BALB/C nude mice (18–20 g) were purchased from the SLAC Laboratory Animal Co (Shanghai, China) and housed under 24–26 °C with a 12 h light/dark cycle with free access to food and water provisions. All experiments and animal procedures were approved by the Ethics Committee of Tongji Hospital, Tongji University School of Medicine.

### Cells lines and cell culture

SKOV3 cells were purchased from the American Type Culture Collection (ATCC; Manassas, VA, USA). RPMI 1640 (Invitrogen, Carlsbad, CA, USA) was used to culture SKOV3 cells after supplementation with 10% fetal bovine serum (Invitrogen). The cells were cultured at 37 °C in 5% CO_2_. For the chemotherapy resistance analysis, SKOV3 cells were pretreated with 0, 0.625, 1.25, 2.5, 5 or 10 μg/ml cis-platinum for 48 h before cell proliferation analysis.

### Transfection of cells with TGF-β1 overexpression or knockdown vectors

TGF-β1 overexpression vectors or vectors containing siRNA against TGF-β1 were constructed by Wuhan Biobuffer Biotech Service (Wuhan, China). TGF-β1 overexpression vectors were constructed by inserting the TGF-β1 sequence into pCDNA3.1 vectors. Vectors with siRNA against TGF-β1 were constructed by inserting a TGF-β1 silencing sequence into pLenR-GPH vectors. TGF-β1 overexpression or downregulation vectors were then transfected into SKOV3 cells at a final concentration of 50 nM using Lipofectamine 2000 (Invitrogen). After transfection for 48 h, SKOV3 cells were collected for the following experiments.

### Cell viability assay

Cell viability was analyzed using the cell counting kit-8 (CCK8; Invitrogen). Briefly, 1 × 10^4^ SKOV3 cells were seeded into a 96-well plate and incubated overnight at the previously described conditions. Then, the medium was discarded and the cells were washed with PBS three times before adding DMEM (90 µl) and CCK8 (10 µl). After incubation for 1.5 h at 37 °C, the optical density (OD) was measured with a microplate reader at 450 nm.

### Western blot

Protein from cells or tissues was extracted standardization with a BCA kit (Pierce, Rockford, IL, USA). Then, protein samples (40 μg) were used for sodium dodecyl sulfate polyacrylamide gel electrophoresis (SDS-PAGE) and transferred to polyvinylidene difluoride (PVDF) membranes. The PVDF membranes were then incubated with the following primary antibodies after blocking in 5% nonfat milk: anti-BRCA1 (1:600), anti-p-Smad3 (1:800), anti-Smad3 (1:800), anti-TGF-β1 (1:1000) and anti-β-actin (1:1000) (Santa Cruz Biotechnology, Santa Cruz, CA, USA). The membranes were then incubated with secondary antibodies (Santa Cruz Biotechnology) for 1 h at room temperature before the chemiluminescence was measured. The Quantity One program (Bio-Rad, Hercules, CA, USA) was used to measure the intensity of the protein bands.

### Quantitative real-time PCR (qRT-PCR)

RNA from the cells was isolated using TRIzol reagent (Molecular Research Center, Cincinnati, OH, USA). Then, cDNA was synthesized using the RNA reverse transcription kit (Invitrogen). The Applied Biosystems 7300 qPCR system (Applied Biosystems; Thermo Fisher Scientific) was used for qRT-PCR analysis. The qRT-PCR reaction conditions were as follows: 95 °C for 10 s, 94 °C for 15 s and annealing at 55 °C for 30 s with 40 cycles. The relative expression level of *TGF*-*β1* was calculated using the 2^−ΔΔCq^ method. The PCR primers used were as follows: *β*-*actin*: forward 5ʹ- AGCGAGCATCCCCCAAAGTT-3ʹ, reverse 5ʹ-GGGCACGAAGGCTCATCATT-3ʹ; *TGF*-*β1*: forward 5ʹ-CAGCAACAATTCCTGGCGATACC-3ʹ, reverse 5ʹ-GCGCTAAGGCGAAAGCCCTCAAT-3ʹ.

### Immunoprecipitation

About 1–4 mg of cell lysates from SKOV3 cells were pre-cleared with protein G beads for 30 min at 4 °C and subsequently incubated with protein G beads prebound with antibody for 2–16 h at 4 °C. The beads were washed with 1% NP40 three times before mixing with sample buffer (6×) and were subjected to SDS-PAGE for western blot detection.

### Tumor xenograft mouse model

Viable SKOV3 cells [5 × 10^6^; containing TGF-β1 overexpression SKOV3 cells, wildtype (control) SKOV3 cells and siRNA-TGF-β1 SKOV3 cells] were subcutaneously injected into nude mice. Tumor sizes were measured every 3 days after subcutaneous injection for 10 days using a vernier caliper (volume = 1/2 × length × width^2^). The mice were sacrificed at 40 days after implantation, the tumors were dissected and TGF-β1, BRCA1 and Smad3 expression in tumor tissues was measured.

### Immunohistochemical analysis

Tumor tissue samples were fixed in 10% formalin, embedded and cut into 5 μm slices. Then, the tumor sections were stained with an anti-TGF-β1 (Santa Cruz Biotechnology) antibody after dewaxing and rehydration. An Axiophot light microscope (Zeiss, Oberkochen, Germany) was used for TGF-β1 expression analysis.

### Statistical analysis

Data are expressed as the mean ± SD. The student’s *t*-test was used to evaluate the differences between groups using SPSS 16.0 (SPSS Inc., USA). A value of *P* < 0.05 was considered a statistically significant difference.

## Results

### Downregulation of TGF-β1 increases chemotherapy sensitivity in ovarian carcinoma cells

**Fig. 1:**
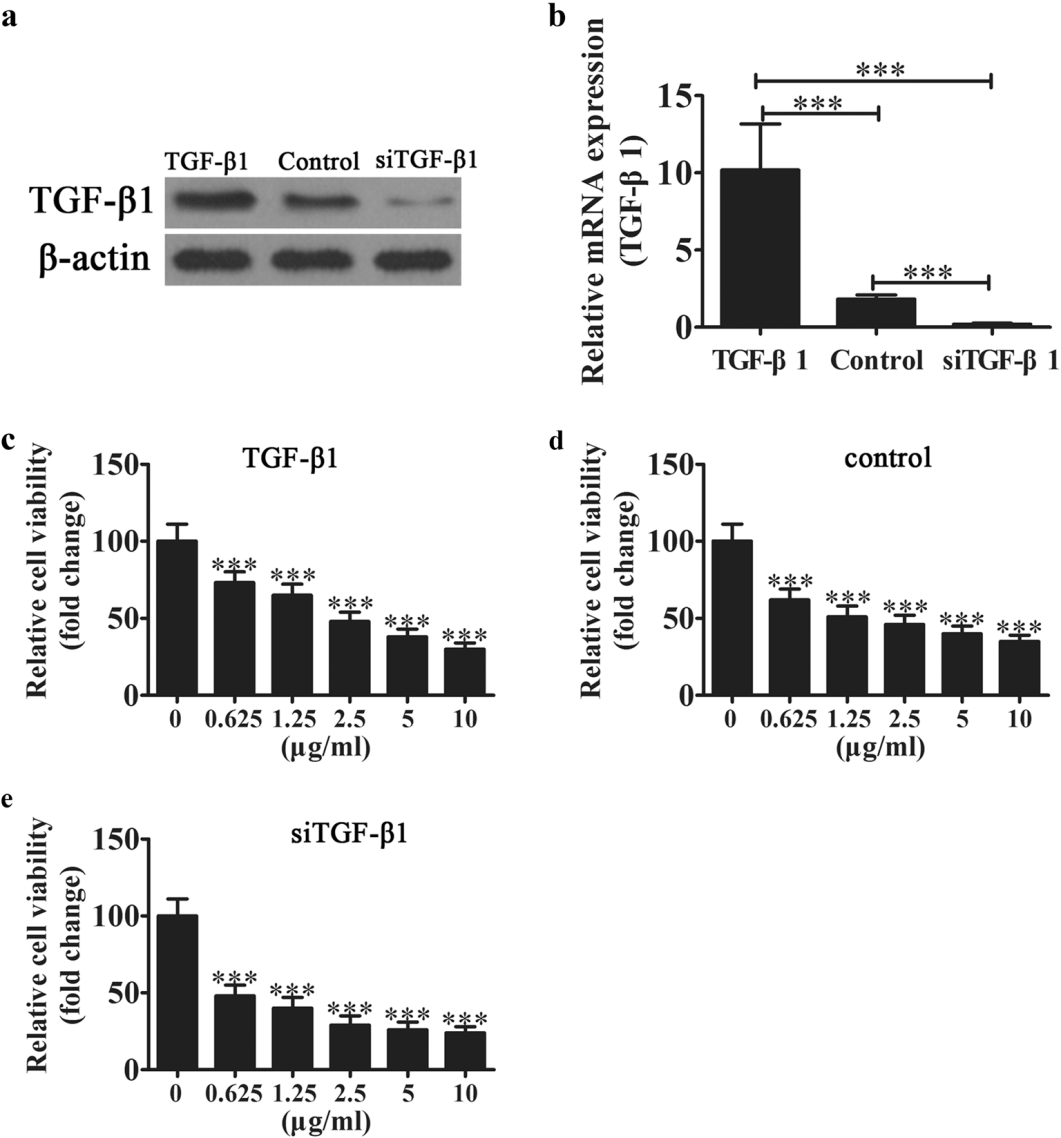
Downregulation TGF-β1 increases chemotherapy sensitivity in ovarian carcinoma cells. Western blot (**a**) and qRT-PCR (**b**) analysis show the expression of TGF-β1 after transfection with TGF-β1 overexpression or siRNA knockdown vectors. Data are expressed as the mean ± SD. **c**–**e** SKOV3 cells were treated with different concentrations of cisplatin for 48 h, and then the cells were collected for CCK8 analysis. Data are expressed as the mean ± SD. ***P < 0.001 versus healthy controls

To identify if TGF-β1 plays a role in OC chemotherapy resistance and proliferation, TGF-β1 overexpression or knockdown vectors were constructed. The results showed that the TGF-β1 level was significantly increased after transfection with the TGF-β1 overexpression vector but was downregulated after transfection with the siRNA vector against TGF-β1 compared with the wildtype control at both the mRNA and protein levels in SKOV3 cells (Fig. 1a, b). The SKOV3 cell line was derived from the ascitic fluid of a Caucasian female with an ovarian tumor. After pretreatment with cisplatin for 48 h. Then, CCK8 assays were used to detect the cell viability of SKOV3 cells. The results showed that knockdown TGF-β1 decreased the chemotherapy resistance of SKOV3 cells, and only 0.625 μg/ml cis-platinum led to a 50% loss in cell viability. Conversely, TGF-β1 overexpression decreased the chemotherapy resistance of SKOV3 cells, leading to a tolerance of 2.5 μg/ml cis-platinum (Fig. 1c–e).

### TGF-β1 knockdown promotes BRCA1/Smad3 signal activation

**Fig. 2:**
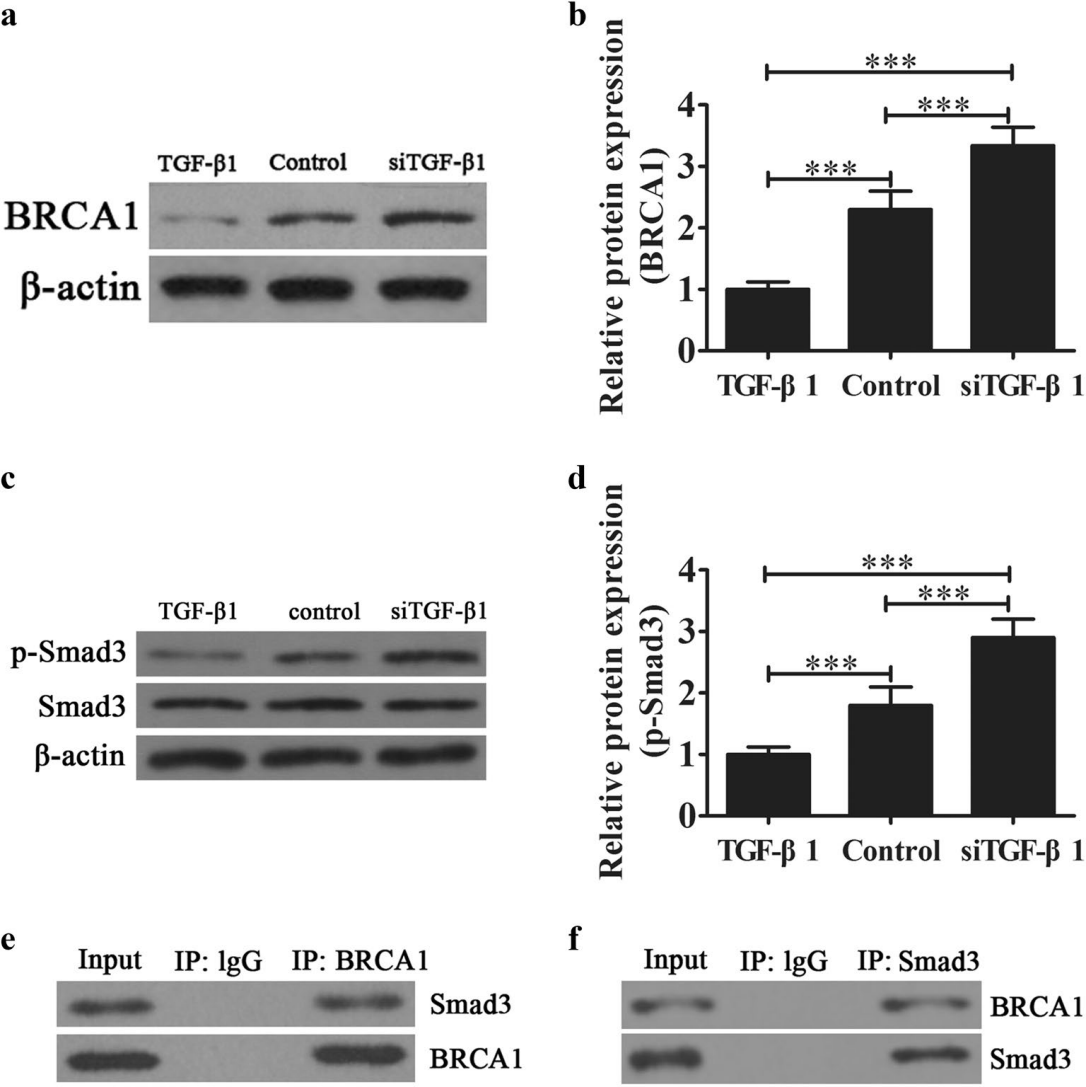
The effect of TGF-β1 on BRCA1/Smad3 signaling. **a**, **b** Western blot showing BRCA1 expression. Data are expressed as the mean ± SD. **c**, **d** Western blot showing the Smad3 expression and phosphorylated Smad3. Data are expressed as the mean ± SD. **e**, **f** Immunoprecipitation analysis of the relationship between Smad3 and BRCA1. ***P < 0.001

Increasing evidence has shown that BRCA1 is involved in the regulation of tumor chemotherapy resistance and proliferation [18, 19]. In our study, western blot analysis showed that dysregulation TGF-β1 decreased BRCA1 expression compared with wildtype SKOV3 cells. However, downregulation of TGF-β1 promoted BRCA1 expression (Fig. 2a, b). Further analysis showed that TGF-β1 knockdown promoted Smad3 phosphorylation. On the other hand, TGF-β1 overexpression decreased Smad3 phosphorylation (Fig. 2c, d). Our co-immunoprecipitation data suggested that BRCA1 can interact with Smad3 (Fig. 2e, f). This suggests that TGF-β1 knockdown increases the chemotherapy sensitivity of SKOV3 cells via promotion of BRCA1/Smad3 signaling.

### TGF-β1 downregulation suppressed tumor growth

**Fig. 3:**
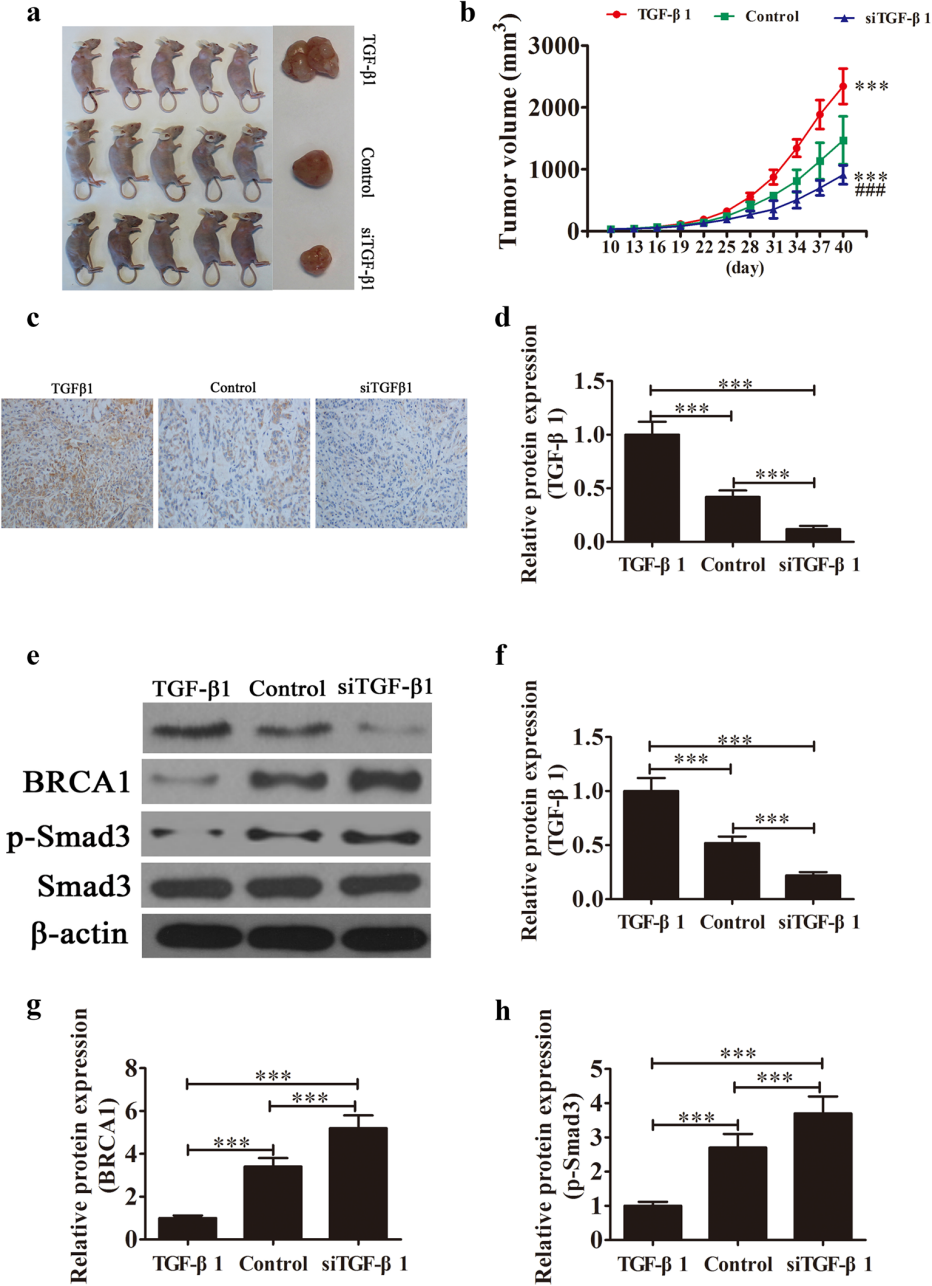
TGF-β1 downregulation suppressed tumor growth of ovarian cancer in a xenograft model. **a** Photographs of tumor tissues from different groups at day 40 (n = 5). **b** Growth curves for tumor volumes in xenografts of nude mice were determined based on the tumor volume measured every 3 days from the tenth day for 40 days (n = 5). ***P < 0.001 versus the control group. ^###^*P* < 0.001 versus the TGF-β1 overexpression group. **c**, **d** Immunohistochemical analysis showing the expression of TGF-β1 in tumor tissues. Data are expressed as the mean ± SD. **e**–**h** Western blot showing the expression of TGF-β1, BRCA1, Smad3 and phosphorylated Smad3. Data are expressed as the mean ± SD. ***P < 0.001

The size of SKOV3 tumors in mice was measured and the results showed that knockdown of TGF-β1 suppressed tumor growth when compared with the control group (Fig. 3a, b). TGF-β1 expression in tumors tissues was then analyzed using immunohistochemistry. The results showed that the TGF-β1 level was increased in the TGF-β1 overexpression group but was decreased in the TGF-β1 knockdown group (Fig. 3c, d). Western blot showed that BRCA1 expression and Smad3 phosphorylation were increased in the TGF-β1 knockdown group. However, upregulation TGF-β1 decreased the level of BRCA1 and Smad3 phosphorylation in tumor tissues (Fig. 3e–h).

## Discussion

Our study reveals a new role and function of TGF-β1 in OC. We show for the first time that knockdown of TGF-β1 suppresses tumor growth and promotes the chemosensitivity of OC cells to cis-platinum, in that we observed a BRCA1 expression increase and the activation of Smad3.

OC is a gynecological disease with a high fatality rate, especially epithelial origin OC (more than 90% of OC cases) [20]. TGF-β1 has been shown to be upregulated in OC and an increasing number of studies has found that in ovarian clear cell carcinomas, the expression of TGF-β1 promotes cancer stem cell properties and the epithelial–mesenchymal transition (EMT) [21]. SMAD transcription factors, which can mediate genetic transcription, are downstream of TGF-β1 [22]. A previous study showed that SMAD3 can be activated by TGF-β1 [23]. Here, we found that TGF-β1 downregulation has effects in addition to the activation of SMAD3. Furthermore, another study found that SMAD3 blocked the phosphorylation of AKT, which promoted the chemosensitivity of hepatocellular carcinoma to cisplatin [24].

In this study, we found that BRCA1 plays an important role in TGF-β1 mediated chemotherapy resistance and tumor growth. We observed that BRCA1 can interact with Smad3 protein, which results in an increase in Smad3-mediated transcriptional activity. Our study also found that BRCA1 mutants suppress the activation of the TGF-β1-responsive reporter or promote Smad3 expression, suggesting that BRCA1 inactivation impairs TGF-β1 signaling [17, 25]. These findings suggest that BRCA1 plays an important role in the link between TGF-β1 and SMAD signaling.

## Conclusion

In conclusion, in this study we show that downregulation of TGF-β1 expression inhibits cell proliferation and increases chemosensitivity in OC by activating BRCA1/Smad3 signaling. TGF-β1-mediated BRCA1/Smad3 signaling may therefore be a novel therapeutic and diagnostic option for OC clinical treatment.

## Abbreviations

TGF-β1: transforming growth factor beta-1
OC: ovarian cancer
EOC: epithelial OC
CCK8: cell counting kit-8
SDS-PAGE: sodium dodecyl sulfate polyacrylamide gel electrophoresis
PVDF: polyvinylidene difluoride
qRT-PCR: quantitative real-time PCR
EMT: epithelial–mesenchymal transition
